# Risk of bias of randomized controlled trials published in orthopaedic journals

**DOI:** 10.1186/1471-2288-13-76

**Published:** 2013-06-09

**Authors:** Laura E Chess, Joel Gagnier

**Affiliations:** 1 Wayne State University School of Medicine, Canfield Dr., Detroit, MI 48201, USA; 2 Department of Orthopaedic Surgery, University of Michigan, Medical Center Dr., Ann Arbor, MI 48109, USA

## Abstract

**Background:**

The purpose of this study was to assess the quality of methodology in orthopaedics-related randomized controlled trials (RCTs) published from January 2006 to December 2010 in the top orthopaedic journals based on impact scores from the Thompson ISI citation reports (2010).

**Methods:**

Journals included *American Journal of Sports Medicine; Journal of Orthopaedic Research; Journal of Bone and Joint Surgery, American*; *Spine Journal;* and *Osteoarthritis and Cartilage*. Each RCT was assessed on ten criteria (randomization method, allocation sequence concealment, participant blinding, outcome assessor blinding, outcome measurement, interventionist training, withdrawals, intent to treat analyses, clustering, and baseline characteristics) as having empirical evidence for biasing treatment effect estimates when not performed properly.

**Results:**

A total of 232 RCTs met our inclusion criteria. The proportion of RCTs in published journals fell from 6% in 2006 to 4% in 2010. Forty-nine percent of the criteria were fulfilled across these journals, with 42% of the criteria not being amendable to assessment due to inadequate reporting. The results of our regression revealed that a more recent publication year was significantly associated with more fulfilled criteria (β = 0.171; CI = −0.00 to 0.342; p = 0.051).

**Conclusion:**

In summary, very few studies met all ten criteria. Thus, many of these studies likely have biased estimates of treatment effects. In addition, these journals had poor reporting of important methodological aspects.

## Background

Randomized controlled trials (RCTs) provide strong evidence for efficacy of healthcare interventions [1]. Carefully planned and well-executed RCTs give us the best estimates of treatment effect and can thus guide clinical decision making [2,3], although trials that lack methodological rigor cause over- or underestimation of treatment effect sizes due to bias [4-6]. Hence, efforts have been undertaken toward improving the design and reporting of RCTs [1,6-11].

While RCTs represent a small proportion of original research published in surgical journals [12,13], they still represent an important component of the literature and a high level of evidence [14]. But, this literature appears to indicate that surgical RCTs lag behind the general literature in terms of methodological quality. Methodological quality mainly refers to the formal aspects of study design, performance and analysis. For example, one study found that only 33% of RCTs published in surgical journals but 75% published in general medicine journals were of high quality [15]. RCTs of orthopaedic surgery appear to be no better, with greater than half of the RCTs in one study lacking proper concealment of randomization, blinding of outcome assessors and reporting of reasons for excluding patients [16]. In another study looking at the quality of RCTs in pediatric orthopaedics, the authors found that only 19% of the included articles met their criteria for high quality [12]. In contrast, it appears that RCTs published in general internal medicine journals is of generally of higher quality. For example, Moher et al. included 211 reports of RCTs from the top four English-language internal medicine journals and found that greater than 60% of RCTs were of high quality [17]. Therefore, it is obvious that RCTs in orthopaedic surgery are in need of improvement.

It is important to note the difference between methodological quality and reporting quality. Our study is designed to evaluate the methodological conduct of studies; however poor reporting can innately make this task difficult. While it is imperative to decipher between reporting and methodology, it can be tempting to draw similar conclusions from both. This will ultimately hamper a true risk of bias assessment and must not be carried out.

To our knowledge, there has not been an assessment of the methodological quality, or risk of bias, of RCTs across the top journals in orthopaedics. Nor has there been an effort to characterize the proportions of published papers that represent the highest levels of evidence. The purpose of the present study was to assess the risk of bias of all randomized trials published in the last 5 years of the top five journals in orthopaedics.

## Methods

### Search strategy

We determined the top five journals in orthopaedic surgery by their impact scores from the Thompson ISI citation reports. These journals included the *American Journal of Sports Medicine* (AJSM), *Journal of Orthopaedic Research* (JOR), *Journal of Bone and Joint Surgery, American* (JBJS Am), *Spine Journal* (SJ) and *Osteoarthritis and Cartilage* (OC). These journals were hand searched on the journal’s website and assessed for reports for inclusion by one individual (LC). Decisions regarding inclusion of potential studies were based on the following criteria: (1) consisted solely of human subjects, (2) random subject allocation, (3) the experimental design included both treatment and control groups comparing an orthopaedic intervention, (4) and had a publication date between January 2006 and December 2010 in the journals mentioned above. These criteria were used as a measure of a methodological quality based Cochrane Collaboration’s widely accepted risk of bias tool as well as Modern Epidemiology 3rd Edition risk of bias assessment recommendations. It is important to note there was no formal protocol for this assessment.

### Data extraction

**Figure 1:**
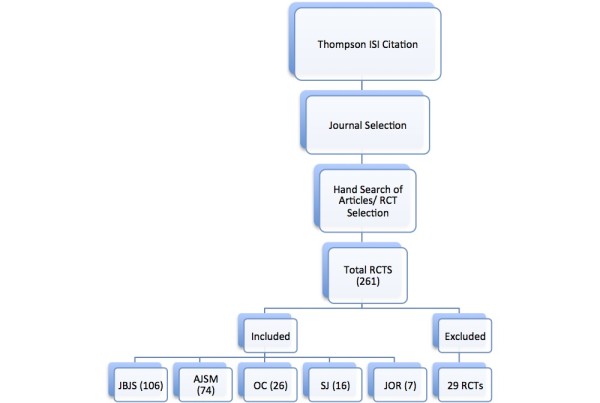
Trial flow diagram.

The investigators separately and independently extracted data from each study using preformatted Excel (Microsoft, Redmond, WA) spreadsheets. Extracted data included: journal name, journal impact factor, and publication year. All included studies were assessed on ten criteria related to risk of bias (Table 1). The ten criteria required sufficient reporting regarding randomization method, allocation sequence concealment, participant blinding, outcome assessor blinding, outcome measurement, interventionist training, withdrawals, intent to treat analyses, clustering, and baseline characteristics. For each of these criteria the RCT was judged as fulfilling each criterion (indicated as a “Yes”), not fulfilling it (indicated as a “No”) or having insufficient information to determine fulfillment (“Not Reported”) (see Figure 1). In order to be considered a “Yes” the paper must have included a complete description regarding the process and outcome of each criterion. If investigators felt that there was too little information or that they would be unable to replicate the process based on unclear reporting, the article was designated as a “Not Reported” for that criterion. A complete lack of reporting or an erroneous method (i.e., Randomization by patient number or date of birth) was marked as “No.” Disagreements were documented and resolved by discussion between data collectors along with the primary investigator.

**Table 1:** Risk of bias criteria

1.	Was the randomization method to groups appropriate?
2.	Was the allocation sequence concealed from those assigning patients to groups?
3.	Were the participants blind to the intervention?
4.	Were the outcome assessors (for the primary outcome) blind to the intervention?
5.	Was the outcome measurement performed in the same manner with similar intensity in the groups being compared?
6.	Were similarly trained individuals administering the intervention across groups?
7.	Were all the withdrawals described?
8.	Were all originally randomized participants analyzed in the groups they were assigned to (i.e., an intention-to-treat analysis)?
9.	Was clustering at the group level accounted for in the analyses?
10.	Were the groups similar at baseline?

### Statistical analysis

Statistical analyses included calculating the mean number of criteria that were met (“Yes), not met (“No”), or of unknown fulfillment (“Not Reported”) within and across all journals. First, we assessed the distribution of Yes/No/NotReported of each article. Then we calculated the mean proportion of fulfilled items for all the articles from the same journals stratified by criterion (Table 2). We then compared these mean proportions across journals using an analysis of variance (ANOVA) to test for differences in reporting quality. To note, the more favorable distribution is one with a greater proportion of fulfilled items, indicating that the journal has met more criteria for methodological quality. Linear regression was also applied with the outcome variable being the total number of fulfilled items per trial and predictor variables being journal impact factor and year of publication. We also performed a sub-analysis on the proportion of met criteria as categorized by geographic location, anatomical region, study size and orthopedic specialty (see Tables 3, 4, 5 and 6). All statistical tests had significance set at p = 0.05.

**Table 2:** Ratings for each methodological quality criterion within and across journals

**Methodological quality criteria**	Journal*	**Yes**	**No**	**Not reported**
Proper Methods of Randomization	AJSM	70.3%	9.5%	20.3%
	JBJS	74.5%	6.6%	18.9%
	JOR	57.1%	0.0%	42.9%
	OC	58.3%	8.3%	33.3%
	SJ	56.3%	0.0%	43.8%
	*Mean*	63.3%	4.9%	31.8%
Concealment of Subject Allocation	AJSM	66.2%	8.1%	25.7%
	JBJS	75.5%	6.6%	17.9%
	JOR	57.1%	0.0%	42.9%
	OC	61.1%	5.6%	33.3%
	SJ	56.3%	0.0%	43.8%
	*Mean*	63.2%	4.1%	32.7%
Blinding of Participants	AJSM	14.9%	18.9%	66.2%
	JBJS	20.8%	23.6%	55.7%
	JOR	14.3%	0.0%	85.7%
	OC	30.6%	16.7%	52.8%
	SJ	25.0%	37.5%	37.5%
	*Mean*	21.1%	19.3%	59.6%
Blinding of Outcome Assessor	AJSM	51.4%	14.9%	33.8%
	JBJS	45.3%	21.7%	33.0%
	JOR	57.1%	0.0%	42.9%
	OC	61.1%	2.8%	36.1%
	SJ	37.5%	18.8%	43.8%
	*Mean*	50.5%	11.6%	37.9%
Outcome Measurements Performed in the Same Manner	AJSM	91.9%	1.4%	6.8%
	JBJS	95.3%	0.9%	3.8%
	JOR	100.0%	0.0%	0.0%
	OC	97.2%	0.0%	2.8%
	SJ	87.5%	0.0%	12.5%
	*Mean*	94.4%	0.5%	5.2%
Interventions Administration by Similarly Trained Individuals	AJSM	79.7%	1.4%	18.9%
	JBJS	48.1%	1.9%	50.0%
	JOR	14.3%	0.0%	85.7%
	Osteo	0.0%	0.0%	100.0%
	SJ	12.5%	0.0%	87.5%
	*Mean*	30.9%	0.7%	68.4%
Description of Compliance With the Intervention	AJSM	77.0%	21.6%	1.4%
	JBJS	64.2%	35.8%	0.0%
	JOR	28.6%	71.4%	0.0%
	OC	83.3%	16.7%	0.0%
	SJ	56.3%	43.8%	0.0%
	*Mean*	61.9%	37.9%	0.3%
Intention-To-Treat Analysis	AJSM	39.2%	2.7%	58.1%
	JBJS	39.6%	1.9%	58.5%
	JOR	14.3%	0.0%	85.7%
	Osteo	63.9%	0.0%	36.1%
	SJ	50.0%	0.0%	50.0%
	*Mean*	41.4%	0.9%	57.7%
Was Clustering Accounted for in the Analysis?	AJSM	0.0%	0.0%	100.0%
	JBJS	0.9%	0.0%	99.1%
	JOR	0.0%	0.0%	100.0%
	OC	0.0%	0.0%	100.0%
	SJ	0.0%	0.0%	100.0%
	*Mean*	0.2%	0.0%	99.8%
Were the Groups Similar at Baseline?	AJSM	74.3%	4.1%	21.6%
	JBJS	71.7%	6.6%	21.7%
	JOR	42.9%	0.0%	57.1%
	OC	63.9%	25.0%	11.1%
	SJ	68.8%	6.3%	25.0%
	*Mean*	64.3%	8.4%	27.3%

*AJSM American Journal of Sports Medicine, *JBJS* Journal of Bone and Joint Surgery, *JOR* Journal of Orthopaedic Research, *OC* Osteoarthritis and Cartilage, *SJ* Spine Journal.

**Table 3:** Sub-group analysis of item responses by anatomical region (article n = 240)

**Randomization**	**Yes (%)**	**No (%)**	**Not reported (%)**
Spine& Neck	14(60.9)	0(0)	9(39.1)
Upper Extremity	32(78.1)	3(7.3)	6(14.6)
Lower Extremity	112(67.9)	14(8.5)	39(23.6)
Mixed	7(70.0)	0(0)	3(30.0)
**Allocation concealment**			
Spine& Neck	14(60.9)	0(0)	9(39.1)
Upper Extremity	32(78.1)	3 (7.3)	6(14.6)
Lower Extremity	111(67.3)	12(7.3)	42(25.5)
Mixed	7(70.0)	0(0)	3(30.0)
**Blinding of Participants**			
Spine& Neck	6(26.1)	7(30.4)	10(43.5)
Upper Extremity	6(14.6)	8(19.5)	27(65.9)
Lower Extremity	35(21.2)	34(20.6)	96(58.2)
Mixed	2(20.0)	2(20.0)	6(60.0)
**Blinding of Outcome Assessors**			
Spine& Neck	11(47.8)	3(13.0)	9(39.1)
Upper Extremity	18(43.9)	10(24.4)	13(31.7)
Lower Extremity	82(49.7)	24(14.6)	59(35.8)
Mixed	7(70.0)	1(10.0)	2(20.0)
**Outcome Measurement**			
Spine& Neck	21(91.3)	0(0)	2(8.7)
Upper Extremity	39(95.1)	0(0)	2(4.9)
Lower Extremity	156(94.6)	2(1.2)	7(4.2)
Mixed	9(90.0)	0(0)	1(10.0)
**Similarly Trained Individuals**			
Spine& Neck	5(21.7)	0(0)	18(78.3)
Upper Extremity	24(58.5)	1(2.4)	16(39.0)
Lower Extremity	81(49.1)	2(1.2)	82(49.7)
Mixed	3(30.0)	0(0)	7(70.0)
**Compliance with Intervention**			
Spine& Neck	13(56.5)	10(43.5)	0(0)
Upper Extremity	29(70.7)	12(29.3)	0(0)
Lower Extremity	119(72.1)	45(27.3)	1(0.6)
Mixed	5(50.0)	5(50.0)	0(0)
**Intent to Treat Analysis**			
Spine& Neck	9(39.1)	1(4.4)	13(56.5)
Upper Extremity	20(48.8)	1(2.4)	20(48.8)
Lower Extremity	70(42.4)	2(1.2)	93(56.4)
Mixed	4(40.0)	0(0)	6(60.0)
**Clustering**			
Spine& Neck	0(0)	0(0)	23(100)
Upper Extremity	0(0)	0(0)	41(100)
Lower Extremity	1(0.6)	0(0)	164(99.4)
Mixed	0(0)	0(0)	10(100)
**Similar at Baseline**			
Spine& Neck	17(73.9)	1(4.4)	5(21.7)
Upper Extremity	30(73.2)	0(0)	11(26.8)
Lower Extremity	117(70.9)	16(9.7)	32(19.4)
Mixed	4(40.0)	3(30.0)	3(30.0)

**Table 4:** Sub-group analysis of item responses by subject

**Randomization**	**Y**	**N**	**DK**
Adult reconstruction	33(71.7)	3(6.5)	10(21.7)
Pediatric Orthopaedic Surgery	5(71.4)	2(28.6)	0(0)
Sports Medicine	50(71.4)	6(8.6)	14(20.0)
Trauma	13(86.7)	1(6.7)	1(6.7)
Foot and Ankle	20(69.0)	2(6.9)	7(24.1)
Hand and Upper Extremity	11(84.6)	0(0)	2(15.4)
Spine	13(56.5)	0(0)	10(43.5)
OA	20(57.1)	3(8.6)	12(34.3)
**Allocation Concealment**			
Adult reconstruction	33(71.7)	3(6.5)	10(21.7)
Pediatric Orthopaedic Surgery	5(71.4)	2(28.6)	0(0)
Sports Medicine	48(68.6)	5(7.1)	17(24.3)
Trauma	13(86.7)	1(6.7)	1(6.7)
Foot and Ankle	20(69.0)	2(6.9)	7(24.1)
Hand and Upper Extremity	11(84.6)	0(0)	2(15.4)
Spine	13(56.5)	0(0)	10(43.5)
OA	21(60.)	2(5.7)	12(34.3)
**Blinding of Participants**			
Adult reconstruction	10(21.7)	9(19.6)	27(58.7)
Pediatric Orthopaedic Surgery	2(28.6)	4(57.1)	1(14.3)
Sports Medicine	10(14.3)	10(14.3)	50(71.4)
Trauma	3(20.0)	5(33.3)	7(46.7)
Foot and Ankle	5(17.2)	9(31.0)	15(51.7)
Hand and Upper Extremity	3(23.1)	2(15.4)	8(61.5)
Spine	5(21.7)	7(30.4)	11(47.8)
OA	11(31.4)	5(14.3)	19(54.3)
**Blinding of Assessors**			
Adult reconstruction	20(43.5)	8(17.4)	18(39.1)
Pediatric Orthopaedic Surgery	3(42.9)	2(28.6)	2(28.6)
Sports Medicine	34(48.6)	10(14.3)	26(37.1)
Trauma	9(60.0)	3(20.0)	3(20.0)
Foot and Ankle	14(48.3)	6(20.7)	9(31.0)
Hand and Upper Extremity	6(46.2)	4(30.8)	3(23.1)
Spine	10(43.5)	3(13.0)	10(43.5)
OA	22(62.9)	1(2.9)	12(34.3)
**Outcome Measurement**			
Adult reconstruction	44(95.7)	1(2.2)	1(2.2)
Pediatric Orthopaedic Surgery	7(100)	0 (0)	0 (0)
Sports Medicine	63(90.0)	1(1.4)	6(8.6)
Trauma	14(93.3)	0(0)	1(6.7)
Foot and Ankle	28(96.6)	0(0)	1(3.5)
Hand and Upper Extremity	13(100)	0(0)	0(0)
Spine	21(91.3)	0(0)	2(8.7)
OA	34(97.1)	0(0)	1(2.9)
**Similarly Trained Individuals**			
Adult reconstruction	25(54.4)	0(0)	21(45.7)
Pediatric Orthopaedic Surgery	3(42.9)	0(0)	4(57.1)
Sports Medicine	54(77.1)	1(1.4)	15(21.4)
Trauma	6(40.0)	1(6.7)	8(53.3)
Foot and Ankle	17(58.6)	1(3.5)	11(37.9)
Hand and Upper Extremity	4(30.8)	0(0)	9(69.2)
Spine	4(17.4)	0(0)	19(82.6)
OA	0(0)	0(0)	35(100)
**Compliance with Intervention**			
Adult reconstruction	32(69.6)	14(30.4)	0(0)
Pediatric Orthopaedic Surgery	5(71.4)	2(28.6)	0(0)
Sports Medicine	53(75.7)	17(24.3)	0(0)
Trauma	9(60.0)	6(40.0)	0(0)
Foot and Ankle	18(62.1)	10(34.5)	1(3.5)
Hand and Upper Extremity	8(61.5)	5(38.5)	0(0)
Spine	12(52.2)	11(47.8)	0(0)
OA	29(82.9)	6(17.1)	0(0)
**Intent to Treat Analysis**			
Adult reconstruction	14(30.4)	1(2.2)	31(67.4)
Pediatric Orthopaedic Surgery	5(71.4)	0(0)	2(28.6)
Sports Medicine	26(37.1)	2(2.9)	42(60.0)
Trauma	6(40.0)	0(0)	9(60.0)
Foot and Ankle	15(51.7)	0(0)	14(48.3)
Hand and Upper Extremity	7(52.9)	0(0)	6(46.2)
Spine	8(34.8)	1(4.4)	14(60.9)
OA	22(62.9)	0(0)	13(37.1)
**Clustering**			
Adult reconstruction	1(2.2)	0(0)	45(97.8)
Pediatric Orthopaedic Surgery	0(0)	0(0)	7(100)
Sports Medicine	0(0)	0(0)	70(100)
Trauma	0(0)	0(0)	15(100)
Foot and Ankle	0(0)	0(0)	29(100)
Hand and Upper Extremity	0(0)	0(0)	13(100)
Spine	0(0)	0(0)	23(100)
OA	0(0)	0(0)	35(100)
**Similar at Baseline**			
Adult reconstruction	33(71.7)	4(8.7)	9(19.6)
Pediatric Orthopaedic Surgery	6(85.7)	1(14.3)	0(0)
Sports Medicine	54(77.1)	3(4.3)	13(18.6)
Trauma	9(60.0)	2(13.3)	4(26.7)
Foot and Ankle	19(65.5)	0(0)	10(34.5)
Hand and Upper Extremity	8(61.5)	0(0)	5(38.5)
Spine	17(73.9)	1(4.4)	5(21.7)
OA	22(62.9)	9(25.7)	4(11.4)

**Table 5:** Sub-group analysis of item responses by Geographic Location

**Randomization**	**Y**	**N**	**DK**
Asia	11(64.7)	4(23.5)	2(11.8)
Europe	78(75.0)	6(5.8)	20(19.2)
N. America	66(62.9)	7(6.7)	32(30.5)
Oceana	9(81.8)	0(0)	2(18.2)
Mixed	1(50.0)	0(0)	1(50.0)
**Allocation Concealment**			
Asia	11(64.7)	4(23.5)	2(11.8)
Europe	78(75.0)	4(3.9)	22(21.2)
N. America	66(62.9)	7(6.7)	32(30.5)
Oceana	8(72.7)	0(0)	3(27.3)
Mixed	1(50.0)	0(0)	1(50.0)
**Blinding of Participants**			
Asia	2(11.8)	0(0)	15(88.2)
Europe	21(20.2)	24(23.1)	59(56.7)
N. America	23(21.9)	24(22.9)	58(55.2)
Oceana	2(18.2)	3(27.3)	6(54.6)
Mixed	1(50.0)	0(0)	1(50.0)
**Blinding of Assessors**			
Asia	9(52.9)	2(11.8)	6(35.3)
Europe	51(49.0)	22(21.2)	31(29.8)
N. America	51(48.6)	13(12.4)	41(39.1)
Oceana	6(54.6)	1(9.1)	4(36.4)
Mixed	1(50.0)	0(0)	1(50.0)
**Outcome Measurement**			
Asia	15(88.2)	1(5.9)	1(5.9)
Europe	99(95.2)	0(0)	5(4.8)
N. America	99(94.3)	1(1.0)	5(4.8)
Oceana	10(90.0)	0(0)	1(9.1)
Mixed	2(100)	0(0)	0(0)
**Similarly Trained Individuals**			
Asia	10(58.8)	0(0)	7(41.2)
Europe	54(51.9)	1(1.0)	49(47.1)
N. America	46(43.8)	2(1.9)	57(54.3)
Oceana	3(27.3)	0(0)	8(72.7)
Mixed	0(0)	0(0)	2(100)
**Compliance with Intervention**			
Asia	11(64.7)	5(29.4)	1(5.9)
Europe	76(73.1)	28(26.9)	0(0)
N. America	69(65.7)	36(34.3)	0(0)
Oceana	9(81.8)	2(18.2)	0(0)
Mixed	1(50.0)	1(50.0)	0(0)
**Intent to Treat Analysis**			
Asia	6(6.1)	0(0)	11(64.7)
Europe	47(45.2)	1(1.0)	56(53.9)
N. America	43(41.0)	3(2.9)	59(56.2)
Oceana	5(45.5)	0(0)	6(54.6)
Mixed	2(100)	0(0)	0(0)
**Clustering**			
Asia	0(0)	0(0)	17(100)
Europe	0(0)	0(0)	104(100)
N. America	1(1.0)	0(0)	104(99.1)
Oceana	0(0)	0(0)	11(100)
Mixed	0(0)	0(0)	2(100)
**Similar at Baseline**			
Asia	13(76.5)	1(5.9)	3(17.7)
Europe	67(64.4)	8(7.7)	29(27.9)
N. America	79(75.2)	9(8.6)	17(16.2)
Oceana	7(63.6)	2(18.2)	2(18.2)
Mixed	2(100)	0(0)	0(0)

**Table 6:** Sub-group analysis of item responses by Study Size (in quartiles)

**Randomization**	**Y**	**N**	**DK**
Size < 51	35(61.4)	5(8.8)	17(29.8)
51 ≤ size < 81	41(70.7)	5(8.6)	12(20.7)
81 ≤ size < 158	53(84.1)	3(4.8)	7(11.1)
Size ≥ 158	36(59.0)	4(6.6)	21(34.4)
**Allocation Concealment**			
Size < 51	33(57.9)	4(7.0)	20(35.1)
51 ≤ size < 81	42(72.4)	5(8.6)	11(19.0)
81 ≤ size < 158	52(82.5)	3(4.8)	8(12.7)
Size ≥ 158	37(60.7)	3(4.9)	21(34.4)
**Blinding of Participants**			
Size < 51	9(15.8)	10(17.5)	38(66.7)
51 ≤ size < 81	9(15.5)	10(17.2)	39(67.2)
81 ≤ size < 158	15(23.8)	19(30.2)	29(46.0)
Size ≥ 158	16(26.2)	12(19.7)	32(54.1)
**Blinding of Assessors**			
Size < 51	25(43.9)	7(12.3)	25(43.9)
51 ≤ size < 81	30(51.7)	10(17.2)	18(31.0)
81 ≤ size < 158	34(54.0)	9(14.3)	20(31.8)
Size ≥ 158	29(47.5)	12(19.7)	20(32.8)
**Outcome Measurement**			
Size < 51	54(94.7)	1(1.8)	2(3.5)
51 ≤ size < 81	54(93.1)	1(1.7)	3(5.2)
81 ≤ size < 158	61(96.8)	0(0)	2(3.2)
Size ≥ 158	56(91.8)	0(0)	5(8.2)
**Similarly Trained Individuals**			
Size < 51	29(50.9)	1(1.8)	27(47.4)
51 ≤ size < 81	38(65.5)	0(0)	20(34.5)
81 ≤ size < 158	31(49.2)	2(3.2)	30(47.6)
Size ≥ 158	15(24.6)	0(0)	46(75.4)
**Compliance with Intervention**			
Size < 51	28(49.1)	29(5.9)	0(0)
51 ≤ size < 81	39(67.2)	18(31.0)	1(1.7)
81 ≤ size < 158	53(84.1)	10(15.9)	0(0)
Size ≥ 158	46(75.4)	15(24.6)	0(0)
**Intent to Treat Analysis**			
Size < 51	10(17.5)	0(0)	47(82.5)
51 ≤ size < 81	15(25.9)	0(0)	43(74.1)
81 ≤ size < 158	39(61.9)	1(1.6)	23(36.5)
Size ≥ 158	39(63.9)	3(4.9)	19(31.1)
**Clustering**			
Size < 51	0(0)	0(0)	57(100)
51 ≤ size < 81	0(0)	0(0)	58(100)
81 ≤ size < 158	1(1.6)	0(0)	62(98.4)
Size ≥ 158	0(0)	0(0)	61(100)
**Similar at Baseline**			
Size < 51	37(64.9)	2(3.5)	18(31.6)
51 ≤ size < 81	39(67.2)	3(5.2)	16(27.6)
81 ≤ size < 158	47(74.6)	7(11.1)	9(14.3)
Size ≥ 158	45(73.8)	8(13.1)	8(13.1)

## Results

We identified a total of 261 RCTs of which 232 met out inclusion criteria. The most common reason for exclusion was the lack of human participants in the RCTs (N = 29). JBJS Am accounted for the largest number of included RCTs (N = 106) followed by AJSM (N = 74), OC (N = 36), SJ (N = 16) and JOR (N = 7). A total of 49% of the criteria were fulfilled across these journals, with 42% of the criteria not being amendable to assessment due to inadequate reporting (Table 7). The RCTs from AJSM had the highest number of fulfilled criteria, or were at the lowest risk of bias, while RCTs from SJ and JBJS Am had the highest number of unfulfilled criteria, and JOR had the largest number of unknown fulfillment of criteria (Table 7). Less than 1% of the included RCTs fulfilled all ten methodological criteria. Results of the ANOVA test revealed that the difference in proportion of items fulfilled (“Yes”) between studies was statistically significant (p = 0.034) at alpha = 0.05 level.

**Table 7:** Summary of overall methodological ratings by journal

Journal title*	**Impact factor**	**Yes†**	**No**	**Not reported**
AJSM (N = 74)	3.80	0.565	0.082	0.353
JBJS (N = 106)	2.97	0.536	0.106	0.358
JOR (N = 7)	2.97	0.386	0.071	0.543
OC (N = 36)	3.95	0.519	0.075	0.406
SJ (N = 16)	3.02	0.450	0.106	0.444
*Mean*		0.491	0.088	0.421

*AJSM American Journal of Sports Medicine, *JBJS* Journal of Bone and Joint Surgery, *JOR* Journal of Orthopaedic Research, *OC* Osteoarthritis and Cartilage, *SJ* Spine Journal.

† ANOVA *P* = .034.

OC had the largest proportion of “yes” ratings, or adequate fulfillment, for four of the ten criteria (proper analysis, description of withdrawals/ compliance, subject blinding, outcome assessor blinding), JBJS Am was the leader for three criteria (randomization process, allocation concealment, accounting for clustering), AJSM led for two criteria (baseline characteristics, intervention administration) and JOR led in one category (blinded outcome assessment). Table 2 contains the complete list of all methodological quality criteria ratings within and across journals.

**Figure 2:**
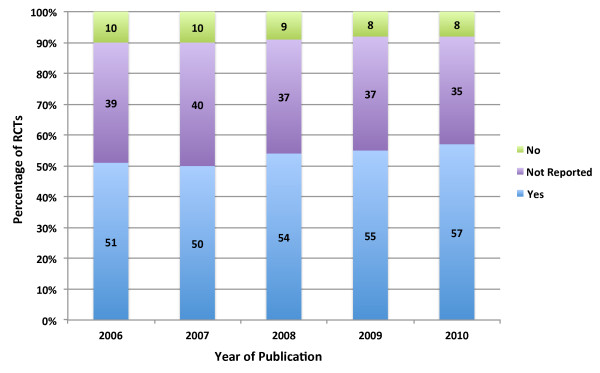
Percentage of RCTs Meeting Criteria by Publication Year.

We also found that the total number of RCTs published increased slightly from 54 in 2006 to 61 in 2008 but fell to 57 and 46 in 2009 and 2010, respectively. But, the proportion of RCTs per total published articles fell from 6% in 2006 to 4% in 2010. The results of our regression revealed that the year of publication was significantly associated with more fulfilled criteria (β = 0.171; CI = −0.00 to 0.342; p = 0.051), but the impact factor was not a significant predictor (β = 0.307; CI = −0.221 to 0.836; p = 0.253). Figure 2 contains the ratings across all criteria by year of publication.

## Discussion

We found that only a very small proportion of the analyzed RCTs met all ten methodological quality criteria, indicating that many of these studies are at a serious risk of bias, but that these trials are improving with time (Figure 2). In addition, we found that many RCTs did not report sufficient information to judge if they met many of the included criteria. Overall, it is clear that the methodological and reporting quality in orthopaedic RCTs has significant room for improvement.

The poor methodological quality of orthopaedic RCTs has been shown in previous literature [12]. Dulai et al. [12] reported that despite increasing numbers of RCTs, only 19% of pediatric orthopaedic trials evaluated met the standard for methodological acceptability. They found that in particular there was inadequate rigor and reporting of randomization methods, use of inappropriate or poorly described outcome measures, inadequate description of inclusion and exclusion criteria, and inappropriate statistical analysis. In another study, Bhandari and colleagues [13] assessed 72 RCTs from JBJS Am published from January 1988 to the end of 2000 and found that while the number of RCTs increased over the years, their mean overall score was only 68.1% of the total Detsky quality score. Similar to our study, they found that more than half of the RCTs were limited by lack of concealed allocation, lack of blinding of outcome assessors, and failure to report reasons for excluding patients [14]. Furthermore, Herman and colleagues [14] found that only 35% of the RCTs in eight leading orthopaedic journals used an intention-to-treat analysis, which was similar to our finding of 41%. Also, Karanicolas and colleagues [15] found that less than 10% of 171 included orthopaedic trauma RCTs had blinded outcome assessors. This is much lower than our nearly 51% finding, the difference of which is most likely due to the broader nature of the trials that we included, going beyond trauma and including any orthopaedic RCTs from a select list of orthopaedic journals.

Beyond methodological deficits in these trials some evidence suggests, similar to our findings, that RCTs in orthopaedic surgery fail to report much important information [16,18]. That is, to adequately assess the quality of any methodological component of an RCT, sufficient information must be present in the published report to make that assessment, and it appears many orthopaedic RCTs fall short in this regard. For example, the most recent of these investigations of reporting quality [19] applied the Consolidated Standards of Reporting Trials (CONSORT) statement [20] to a sample of RCTs, the Strengthening the Reporting of Observational Studies in Epidemiology (STROBE) statement [21] to a sample of case–control, cohort and cross-sectional studies, and a statistical questionnaire was used to assess all included studies. They found that for the 100 included studies only 58% of the CONSORT items were met on average across the seven included journals. We found inadequate reporting on average for approximately 42% of the items on which the RCTs were assessed. The slight difference in findings between these studies can likely be accounted for by the use of different checklist items and the inclusion of different selections of journals. Either way, research in the area indicates serious inadequacies in reporting in orthopaedic RCTs. This trend of poor reporting has been seen in other fields as well, including internal medicine [17] and general surgery [22].

Despite the deficits, the RCTs we included did have some common strengths. In general, the intervention and primary outcome was well described in most papers. Also, the proportion of methodological quality items fulfilled increased with increasing publication year, which is consistent with trends in internal medicine journals [17]. This is promising and may suggest that clinical trialists, editors and reviewers are putting more emphasis on proper methodology.

Our study has several strengths. First, we conducted a comprehensive hand search of the tables of contents of the top orthopaedic journals in a recent span of 5 years. Thus, the findings presented here for the included RCTs likely represent the most read and cited RCTs in the orthopaedic community and therefore give an excellent idea of the quality of the RCTs that might be impacting clinical decision making. If in fact this assumption is true, the trials that are the most influential are at a high risk of having biased estimates of treatment effect. But, due to the limited selection of journals included, it is possible that higher quality and more influential RCTs are being published in other journals. For example, we found that RCTs make up only a very small proportion of all articles published in these five journals and therefore may not be influencing decision making to any high degree. In order to ensure a proper meta-analysis, our paper is in accordance with the PRISMA Statement and meets all criteria. Additionally, we included methodological quality criteria that have been empirically proven to bias estimates of treatment effect when not properly implemented [3,4,23-34]. All included criteria have empirical evidence that not using them in RCTs or not assessing them in systematic reviews results in bias in the estimates of treatment effect or in misclassification of trials as high or low quality. But, due to the lack of reporting of the included studies we could not directly test the influence of specific inadequacies in methodology on effect estimates. Therefore, we cannot be certain that the flaws in methodology in these orthopaedic studies absolutely bias the estimates of treatment effect. We can only extrapolate for the extensive literature that has shown this to be true for RCTs in other clinical areas [3,4,23-34].

It is important to note that just because a study did not report a certain methodology does not imply that it was not performed. For example, in this study, subject allocation and cluster analysis had two of the lowest fulfillment proportions. We acknowledge that descriptive reporting of these topics may not have been emphasized despite proper methodology and that poor reporting may not necessarily be a proxy for poor methodology [35]. Thus, this paper fails to account for these underreporting deficiencies and may falsely underestimate the quality of methodology in this literature. In any case, to adequately assess the quality of a reported study the relevant information must be present for the reader to assess the potential risk of bias in the estimates of effect to determine the potential import or not of the RCT to clinical decision-making.

In common with other authors, we can make some recommendations on how to improve this literature. First, we suggest that investigators include on their team an epidemiologist, clinical epidemiologist, clinical trial methodologist or someone with experience in conducting RCTs and a statistician or biostatistician to ensure proper planning and implementation of the trial. There is evidence that including such individuals on the investigative team improves the quality of the resultant RCT [13]. In addition, we would suggest that investigators and authors refer to the revised CONSORT statement [20] and the related explanatory paper [36] to guide them on the important information to include when reporting their RCT. The CONSORT statement has been shown to improve the quality of reporting in these studies [37]. In addition to these documents, there are other reporting guidance documents located on the equator network website that may be of use [11]. Finally, we suggest that journal editors enforce the use of the CONSORT statement so that published reports are completely reported and have the best chance of being interpreted properly for clinical decision making.

## Conclusions

There are some obvious flaws in the methodology and reporting of RCTs in the orthopaedic literature. These flaws may cause seriously biased estimates of effect in those studies. We expect that these types of initiatives mentioned above will improve these important types of clinical research which are an integral aspect to improving the empirical base for orthopaedic procedures [38]. And remember, just because a study is rated as level I evidence does not imply that it is without methodological flaws and that these flaws can bias the reported effect estimates [39].

## Competing interests

The authors declare that they have no competing interest.

## Authors’ contributions

JG designed the study, performed statistical analysis, and revised the manuscript. LC carried out the data collection, participated in the statistical analysis, drafted the manuscript and designed the figures/tables. Both authors read and approved the final manuscript.

## Pre-publication history

The pre-publication history for this paper can be accessed here:

http://www.biomedcentral.com/1471-2288/13/76/prepub
